# Oncodomains: A protein domain-centric framework for analyzing rare variants in tumor samples

**DOI:** 10.1371/journal.pcbi.1005428

**Published:** 2017-04-20

**Authors:** Thomas A. Peterson, Iris Ivy M. Gauran, Junyong Park, DoHwan Park, Maricel G. Kann

**Affiliations:** 1 Department of Biological Sciences, University of Maryland, Baltimore County, Baltimore, Maryland, United States of America; 2 University of California, San Francisco, Institute for Computational Health Science, San Francisco, California, United States of America; 3 Department of Mathematics and Statistics, University of Maryland, Baltimore County, Baltimore, Maryland, United States of America; Center for Cancer Research, UNITED KINGDOM

## Abstract

The fight against cancer is hindered by its highly heterogeneous nature. Genome-wide sequencing studies have shown that individual malignancies contain many mutations that range from those commonly found in tumor genomes to rare somatic variants present only in a small fraction of lesions. Such rare somatic variants dominate the landscape of genomic mutations in cancer, yet efforts to correlate somatic mutations found in one or few individuals with functional roles have been largely unsuccessful. Traditional methods for identifying somatic variants that drive cancer are ‘gene-centric’ in that they consider only somatic variants within a particular gene and make no comparison to other similar genes in the same family that may play a similar role in cancer. In this work, we present oncodomain hotspots, a new ‘domain-centric’ method for identifying clusters of somatic mutations across entire gene families using protein domain models. Our analysis confirms that our approach creates a framework for leveraging structural and functional information encapsulated by protein domains into the analysis of somatic variants in cancer, enabling the assessment of even rare somatic variants by comparison to similar genes. Our results reveal a vast landscape of somatic variants that act at the level of domain families altering pathways known to be involved with cancer such as protein phosphorylation, signaling, gene regulation, and cell metabolism. Due to oncodomain hotspots’ unique ability to assess rare variants, we expect our method to become an important tool for the analysis of sequenced tumor genomes, complementing existing methods.

## Author summary

The analysis of somatic variants in sequenced tumor samples is important for understanding the molecular disruptions that underlie the vast differences in individual cancer phenotypes or response to treatment. In order to understand which somatic mutations are functionally important for the initiation or progression of cancer, traditional analyses are ‘gene-centric’ in that they focus on single genes with high mutation frequency in tumor samples. However, many genes with experimental evidence of cancer involvement are found to be mutated in only a few tumor samples, hampering the data-driven identification of important genes. In our analysis, we leverage decades of important findings from structural genomics into the study of somatic variants by utilizing conserved protein domain families. Our method identifies ‘oncodomain hotspots’, sites within protein domain families with high mutation frequency in tumor samples. This enables our method to assess the importance of even rare variants by comparing to other genes with the same protein domain. By incorporating the structural and functional context encapsulated in protein domain families, we can identify even rare somatic variants in 5,437 genes, 3,041 of which are novel gene associations to cancer but are similar in structure and/or function to known cancer genes.

## Introduction

In recent years, studies analyzing sequenced tumor genomes have seen a drastic increase in their sample sizes, growing from only a handful samples to cohorts of several thousand patients. This rise in availability of sequenced tumor samples has enabled the comparative analysis of tumors originating from different tissues, revealing a diverse tissue-specific genomic landscape of mutational patterns [1–6]. Revelations of this complexity observed in sequenced tumor samples has led to new insights into cancer genomics. However, identifying which somatic variants are the “drivers” behind initiation or progression of cancer is confounded due to the high prevalence of “passenger” mutations that occur with low frequency but are thought to have no functional effect [7,8]. Thus, despite the increase in tumor-derived data, we are unable to understand whether the vast majority of somatic variants in tumor samples have any functional role.

Towards understanding which somatic variants influence the initiation or progression of cancer, much work has been devoted to the cataloging of sequencing data in repositories like the Catalog of Somatic Variants in Cancer (COSMIC) [9] and to manually curated lists of genes with evidence of cancer involvement in GeneCards [10], the Cancer Gene Census (CGC) [11], the NCI Cancer Gene Index [12], the “proto-oncogene” and “tumor suppressor” classifications in the UniProt [13] database, the Network of Cancer Genes [14], and the TSGene database [15]. Massive ongoing sequencing projects like The Cancer Genome Atlas (TCGA) have discovered thousands of genes that are mutated in only a small fraction of tumors yet may still be important for cancer initiation or progression [7,16–18]. This has led to a rise in the availability of tools for analyzing and visualizing data [19–23] and also for identifying genes in tumor samples that are likely to harbor somatic variants that drive cancer initiation or progression [1,2,24,25]. Traditionally, methods for identifying important genes in tumor samples identify genes that are significantly enriched with somatic variants by clustering somatic variants by genes for statistical analysis. Clustering variants by gene regions is the natural choice since genes are units of inheritance and much is known about the function of particular genes. Not surprisingly, gene-centric studies of TCGA data have been able to recapitulate much of the knowledge about cancer genetics derived from decades of studies [1,2,6,24,25]. For instance, methods like the Cancer Mutation Prevalence Score (CaMP Score) in Sjöblom *et al*. [1], Wood *et al*. [2], and MutSigCV in Lawrence *et al*. [24] employ frequency-based analyses to identify regions of the genome (i.e., genes) that contain more mutations than expected by chance given a background of randomly occurring passenger mutations. However, the gene-centric analysis of individual cancer data relies on the relative frequency of all variants in a gene in sequenced tumor samples and is likely to miss variants that influence cancer progression that occur with relatively low frequency in the population. Even in the early years of such gene-centric data-driven analyses of sequenced tumor genomes like the CaMP Score, it was discovered that the genomic landscapes of somatic mutations in cancer were dominated by ‘gene hills’, or gene regions that are mutated at a low frequency. Indeed, it has been shown that even well-studied genes in cancer are mutated in only a small portion of tumor samples [18,26]. Thus, to identify infrequently mutated genes that play a role in cancer progression, other methods have been developed for clustering low frequency gene-mutations together with other genes with a common functional role. For example, clustering variants from genes on the same pathway [24,27–30], ontological term [28,31], or protein interacting partners [32,33]. Additionally, akin to tools for predicting deleterious variants in other diseases, machine learning methods [34–36] have been developed to determine which variants are likely to influence cancer progression. For instance, the Cancer-specific High-throughput Annotation of Somatic Mutations (CHASM) [34], is a machine learning predictor trained to classify between variants known to drive cancer progression and putatively neutral variants using properties of genomic and protein sequence, predicted protein structure, and multiple sequence alignments.

In recent work, Nehrt *et al*. [37] and Yang *et al*. [38] have shown the value of analyzing cancer somatic variants by clustering variants within a gene sub-region, i.e., the protein domain. Protein domains are the functional, structural, and evolutionary units of proteins [39,40], mediate approximately 75% of protein-protein interactions [41], and mutations in different domain regions of the same gene can have functionally and phenotypically distinct effects [42]. So, protein domain level studies have shown great potential to analyze tumor variants, in particular because they overcome the inability to distinguish functionally relevant variants due to the modularity and polyfunctionality of genes. In their domain-centric studies, somatic variants from TCGA of two [37] and later twenty [38] tumor types were analyzed to identify specific domain regions within genes that are significantly mutated in somatic tumor samples. In Nehrt *et al*., it was discovered that domain regions within a single gene can display heterogeneous mutation patterns that are unique between Breast Invasive Carcinoma and Colorectal Adenocarcinoma. Extrapolated to the plethora of cancer types available in the TCGA project, Yang *et al*. further defined these unique domain mutational patterns, highlighting patterns specific to any of these cancer types. In these previous domain-centric analyses, statistical measures were performed to identify domain families that are frequently mutated often with mutations from multiple genes with a common protein domain. In this work, we develop a novel method to identify “oncodomains”, or protein domains in which somatic variants from one or more genes encoding the domain occur more frequently at specific sites (i.e., oncodomain hotspots) than expected by chance. These oncodomain hotspots correspond to specific positions within an entire family of genes, which enables our method to study even extremely rare somatic variants via inference to other genes with similar somatic variant patterns. We argue that since protein domains are the structural and functional units of proteins, protein domains are the ideal framework for comparison to other genes since they are manually curated to match the structure and known functional features of domain family members, providing an inherent functional explanation of how somatic variants can contribute to cancer. To clarify, the approaches by Nehrt *et al*. and Yang *et al*. identified domain families that were enriched with somatic mutations but they did not, however, analyze the position-specific mutational patterns between different genes that share a common protein domain as in this work. The oncodomain concept introduced here is motivated by results from our earlier studies on known disease mutations. In Peterson *et al*. [43–45], we performed a domain-centric study to cluster all known disease variants into common domain regions from all human proteins. Results from these studies hinted at protein domain positions of functional relevance for the analysis of variants from the OMIM [46] and Swiss-Prot [47] databases. Specifically, known disease variants tend to cluster at specific domain sites more than expected by chance and these ‘position-based domain hotspots’ tended to be located on functional features and conserved residues, properties that were also found for variants that have been experimentally determined to be phenotypically altering in yeast [45]. Here we tested the hypothesis of whether cancer somatic variants also present similar patterns of aggregation as known disease variants. To address this question, we developed a new statistical framework in which we control for population-level frequency information and the large proportion of cancer passenger mutations. Oncodomain hotspots are derived exclusively from somatic mutations from sequenced tumor samples and represent a novel approach for assessing which somatic mutations are likely to influence the initiation or progression of cancer.

Although domain-centric models have been previously developed in Nehrt *et al*., Yang *et al*., the oncodomain method differs in substantial ways. Firstly, these studies were region-based in that entire domain regions were assessed for cancer significance, not specific positions within the domain family. Although Yang *et al*. identifies mutational hotspots, these hotspots are specific to a particular gene and contain no information from other genes sharing a common protein domain. Furthermore, the hotspots in Yang *et al*. do not consider variants from all domain regions as they restrict their analysis to domains that are significant in their region-based model. Secondly, oncodomains are inherently family-based in that somatic variants are aggregated to the domain-level and significance of a specific family member is ascertained by referencing all members of the family. Although Nehrt *et al*. analyzed domain regions from all genes sharing a common domain, the regions were concatenated and treated as a single, large gene and thus no positional information was used. Thirdly, the study conducted by Yang *et al*. only considers somatic variants that are predicted to be “potentially damaging” via the IntOGen-mutation platform [48] and removes all other somatic variants from the analysis. The IntOGen-mutation platform is a meta-predictor that classifies variants as “potentially damaging” primarily on the observed frequency in tumor samples and the results of several variant predictors, SIFT [49], PolyPhen-2 [50], VEP [51], and MutationAssessor [52]. This contrasts with oncodomain hotspots, which consider all somatic variants no matter the observed frequency and does not utilize machine learning methods to remove variants predicted to have no functional impact. Notably, filtering the data using variant predictors is problematic since it will bias the remaining variants towards conserved sites, functional features, structurally important residues, and even domain regions since this information is used in the variant predictors to assess deleteriousness.

In this work, we compare the results of oncodomain hotspots to genes with evidence of cancer involvement from the Cancer Gene Census, the NCI Cancer Gene Index, the Network of Cancer Genes, TSGene, and UniProt and to mainstream methods for the classification of cancer variants from tumors. Specifically, we compared to a gene-centric method, MutSigCV, two domain-centric approaches developed by Nehrt *et al*. and Yang *et al*., and a multi-feature machine learning predictor trained to distinguish drivers from passengers, CHASM. We demonstrate that oncodomain hotspots not only overlap well with the cancer genomics literature and the results of both gene- and domain-centric methods, but also that our method is unique in the ability to detect variants that occur with low frequency in tumor samples but have evidence of cancer involvement or are predicted to be driver mutations by CHASM. Due to the ability of oncodomain hotspots to leverage relevant structural and functional context to identify even rare somatic variants with high potential to drive cancer development, we hope for oncodomain hotspots to become an important tool for large-scale analysis of sequenced somatic tumor samples, complementing existing tools.

## Materials & methods

### Mapping somatic variants to specific protein domain positions

**Fig 1:**
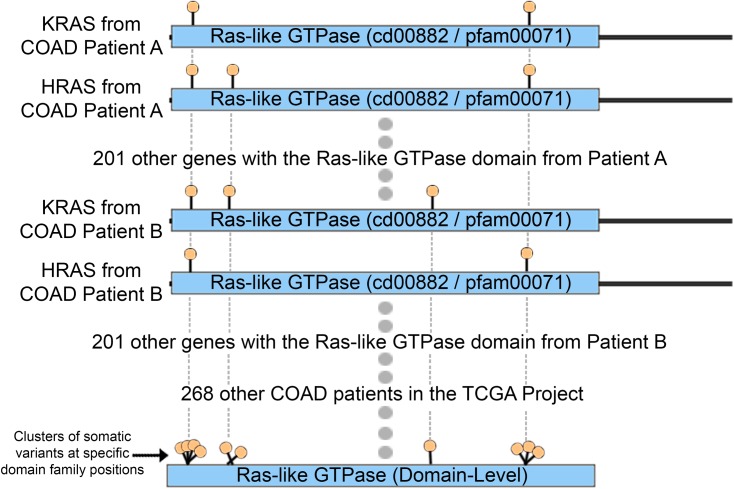
Depiction of the process of mapping variants to domain positions to find oncodomain hotspots.

Somatic Variants from 5,848 patients from The Cancer Genome Atlas (TCGA) [53] were mapped to specific positions within protein domain models to identify clusters. TCGA MAF files were obtained on July 7^th^, 2014 for 20 cancer types: Adrenocortical Carcinoma (ACC), Bladder Urothelial Carcinoma (BLCA), Brain Lower Grade Glioma (LGG), Breast Invasive Carcinoma (BRCA), Colon Adenocarcinoma (COAD), Glioblastoma Multiforme (GBM), Head and Neck Squamous Cell Carcinoma (HNSC), Kidney Chromophobe (KICH), Kidney Renal Clear Cell Carcinoma (KIRC), Liver Hepatocellular Carcinoma (LHIC), Lung Adenocarcinoma (LUAD), Lung Squamous Cell Carcinoma (LUSC), Ovarian Serous Cystadenocarcinoma (OV), Pancreatic Adenocarcinoma (PAAD), Prostate Adenocarcinoma (PRAD), Rectum Adenocarcinoma (READ), Skin Cutaneous Melanoma (SKCM), Stomach Adenocarcinoma (STAD), Thyroid Carcinoma (THCA), and Uterine Corpus Endometrial Carcinoma (UCEC). Only validated exonic variants were used, resulting in 1,326,954 unique exonic variants across 20 cancer types. The number of patients and variants for each of the 20 cancer types studied is enumerated in Table 1. To map protein domain models to specific positions within human proteins, a human protein database containing 54,372 proteins was created with 33,963 proteins from RefSeq [54] and 20,409 proteins from Swiss-Prot [55] downloaded via NCBI’s E-utilities [56]. Since redundant protein entries exist between the RefSeq and Swiss-Prot databases, we selected only one representative protein for each unique Entrez gene ID, either the longest Swiss-Prot protein, or the longest RefSeq protein if no Swiss-Prot protein was listed for the gene ID. In addition, to avoid redundancy between isoforms produced by a single gene, we used only the longest protein product for analysis. Protein domain models from CDD [57] and Pfam [58] were obtained from the Conserved Domain Database (CDD version 2.25). HMMer’s semi global implementation [59] was used to map these domain models from human proteins. Finally, illustrated in Fig 1, proteins with somatic variants were aligned to specific positions within each domain model by using HMMer’s alignment with an E-Value threshold ≤ 0.001 where variants on gap regions of the domain model were assigned to the last position before the gap. To build the CDD protein domain set with minimal redundancy on the models, we selected only root domains (obtained from ftp://ftp.ncbi.nlm.nih.gov/pub/mmdb/cdd/cdtrack.txt). The final domain sets that map to human proteins contain 4,377 and 4,118 protein families from CDD and Pfam respectively.

**Table 1:** Number of patients, somatic variants, oncodomains, and oncodomain hotspots for each cancer type.

Cancer Type	Number of Patients	Number of Exonic Somatic Variants	Number of Pfam Oncodomains (*fdr*(*t*) = 0.05)	Number of Pfam Oncodomains (*fdr*(*t*) = 0.01)	Number of Pfam Oncodomain Hotspots (*fdr*(*t*) = 0.05)	Number of Pfam Oncodomain Hotspots (*fdr*(*t*) = 0.01)
ACC	91	41,451	44	42	67	62
BLCA	130	39,312	31	17	73	31
BRCA	977	90,490	68	41	255	123
COAD	270	125,522	148	108	646	435
GBM	291	22,166	32	21	69	47
HNSC	306	74,008	4	3	7	6
KICH	66	3,835	1	1	1	1
KIRC	422	55,092	52	36	139	70
LGG	289	14,817	20	17	45	33
LIHC	202	92,840	74	49	364	182
LUAD	542	255,972	142	77	1,550	1212
LUSC	138	49,997	30	21	210	116
OV	375	21,207	16	10	49	43
PAAD	91	46,505	41	29	73	42
PRAD	259	9,437	7	6	18	11
READ	116	34,259	52	31	106	67
SKCM	344	290,341	345	200	1,742	1,258
STAD	289	148,520	119	78	828	574
THCA	402	7,458	8	7	12	7
UCEC	248	240,546	258	161	1,547	1,186

### Identifying cancer-specific oncodomain hotspots within protein domain families

In previous work by Peterson *et al*. [43,44] and Yue *et al*. [60] it was shown that variants with known cancer relevance from the OMIM and UniProt databases tend to cluster at positions within protein domains. However, the inclusion of patient frequency information is critical for the analysis of TCGA somatic variants from sequenced tumor samples and for the identification of driver mutations, but requires a new statistical framework that includes patient frequency into the analysis. Thus, in this work, we developed a mutational score to classify protein domain positions derived from individual patient data using a local false discovery rate (FDR) with a Zero-Inflated Poisson (ZIP) null distribution. We applied this methodology separately for each cancer type and for each protein domain model and defined high scoring protein domain positions as those with a q-value < 0.05. The details and derivation of this statistical approach can be found in a separate work by Gauran *et al*. [61] but briefly, the formulation used is as follows.

At the protein domain-level which often encompasses several genes, each position within the domain contains *j* = 0,1,…*j*_*max*_ somatic mutations from patients with the same cancer type and we define *n*_*j*_ as the number of domain positions with *j* somatic variants. We developed a local false discovery rate method using a zero-inflated Poisson distribution as the null distribution for non-significantly mutated positions. Each protein domain was considered separately to remove the influence of region-based cofactors (replication timing, expression, etc.) since each domain position is aligned to the same set of proteins. Our goal is to find the cutoff of *j* which separates non-significantly (*f*_0_(*j*)) and significantly (*f*_1_(*j*)) mutated positions. The observed count of mutations are from a mixture distribution, where

$$p_{0}h=\mathrm{Pr}(\mathrm{non-significant})$$

$$p_{1}h=\mathrm{Pr}(\mathrm{significant})$$

$$f_{0}(j)=\mathrm{density}\;\mathrm{if}\;\mathrm{non-significant}$$

$$f_{1}(j)=\mathrm{density}\;\mathrm{if}\;\mathrm{significant}$$

Where *f*_0_ is assumed to follow a Zero Inflated Poisson (ZIP) distribution while *f*_1_ could be any other (discrete) distribution. ZIP models are considered useful for the analysis of count data with a large amount of zeros because it allows for two sources of overdispersion by mixing a Poisson distribution with zero-inflation. For a given position, we assume that the number of mutations *j* is generated by one of the two distributions *f*_0_(*j*) or *f*_1_(*j*) so the probability density function of the mixture distribution is

$$f(j)=p_{0}f_{0}(j)+p_{1}f_{1}(j)$$

Then, we define the local FDR at *t* as

$$fdr(t)=\frac{p_{0}f_{0}(t)}{f(t)}$$

Which indicates that *f dr*(*t*) is the posterior probability that a position with *j* = *t* is non-significant. The interpretation of the local FDR value is analogous to the frequentist’s p-value wherein local FDR values less than a specified level of significance provide stronger evidence against the null hypothesis. In this work, unless noted otherwise, we use a cutoff of *f dr*(*t*) = 0.05, which would indicate that only 5% our oncodomain hotspots are false discoveries.

When comparing regions of the genome (i.e., genes in the CaMP score and MutSigCV), methods must account for “covariates” that are thought to influence the background rate of passenger mutations for that particular genomic region, such as replication timing, gene expression, chromatin state (open/closed), and mutation context (e.g., C to G in CpG sites, G to C in GpA sites, etc.). When analyzing an aligned position within the same family of genes, the altered mutation rate of the aligned gene regions does not differ between aligned positions and thus does not need to be modeled. This is correct for all covariates with the exception of mutational context, which may differ between aligned positions. However, we determined that using synonymous variants to estimate the background probability of passenger mutations was inappropriate. Firstly, it is well known that many synonymous variants are drivers that re-occur in cancer and are not distributed randomly [62–64]. Secondly, the frequency of occurrence of synonymous variants is often different than that of the nonsynonymous variants, making them inappropriate to use to estimate the null model. Thus, using a randomly distributed background of equal size to the observed nonsynonymous variants was chosen.

### Overlap with functional features and conserved positions

To assess the significance of overlap between oncodomain hotspot positions and positions that have known function, functional feature annotations for each protein position were obtained from UniProt on July 18^th^ 2015. To determine the conservation of each domain position *j*, we employed the AL2CO [65] algorithm for assessing entropy via the following formula:

$$H_{j}h=-\sum_{i=1,20}p(a_{i},{}_{j})\ln(p(a_{i},{}_{j}))$$

Here, *p*(*a*_*i*,*j*_) is the amino acid frequency for amino acid *a*_*i*_ at position *j* and *H*_*j*_ is the AL2CO score at position *j*. Positions were considered to be conserved if they were greater than or equal to the average AL2CO score plus one standard deviation. Pearson’s correlation coefficient and Fisher’s exact test with Bonferonni correction were used to assess significance of hotspot position overlap with functional features or conserved residues.

### Comparison to other methods & cancer-related databases

To compare to other methods, significantly mutated genes were obtained using MutSigCV v1.4, significantly mutated domains were obtained from the results of Nehrt *et al*. and Yang *et al*., and the results of CHASM were obtained from the Firehose project [19]. To compare to cancer-related databases, the Gene Ontology database [66] along with the pfam2go annotations were obtained on August 21^st^ 2015, the NCI Cancer Gene Index was obtained on March 7^th^, 2016, the Network of Cancer Genes was obtained on March 4^th^, 2016, and the TSGene database was obtained on March 4^th^, 2016, the Cancer Gene Census [11] on November 6^th^, 2015, and the UniProt [13] “proto-oncogene” and “tumor suppressor gene” classifications were obtained on November 7^th^, 2015. Gene Ontology category enrichment was performed using Fisher’s exact test with Bonferroni correction.

## Results

### Oncodomains and cancer-specific oncodomain hotspots

In this work, we define oncodomains as families of protein domains in which somatic variants from one or more genes containing the same domain form a hotspot. Oncodomain hotspots are defined as protein domain positions where somatic variants for a specific cancer type occur more frequently than expected by chance (see Materials & Methods). A comparison of the number of oncodomains and oncodomain hotspots identified for different *fdr(t)* cutoffs along with the number of patients and exonic somatic variants for each cancer type is shown in Table 1. For simplicity, we will refer to the results obtained using the *fdr(t)* cutoff of 0.05 for the remainder of this analysis. In this study, we identify 185 protein domain families from CDD and 673 from Pfam across 20 cancer types as oncodomains. Within these families, 2,126 oncodomain hotspots were identified on CDD domains and 3,563 hotspots were identified on Pfam domains. Overall, the quantity and location of the hotspots were found to be highly heterogeneous between cancer types. We find the number of oncodomains and oncodomain hotspots to be highly variable between cancer types ranging from only 1 or 7 hotspots in KICH and HNSC respectively, to a maximum of 1,742 hotspots identified in SKCM. In our dataset, TCGA cancer types had an average of 74 (standard deviation of 89.8) oncodomains and an average of 309 (standard deviation of 571) oncodomain hotspots. The frequency of hotspots across the 20 cancer types was highly heterogeneous with nearly 400 domain models being signatures for only one cancer type while 21 were common to ten or more cancer types (S1 Fig & S1 File). A full list of all oncodomains and the cancer-specific oncodomain hotspots for each cancer type can be found in S2 File.

We find a strong correlation between the total number of exonic somatic variants and the number of oncodomains / oncodomain hotspots (Pearson’s Correlation 0.92 and 0.98 respectively). Compared to the number of exonic variants, the number of patients in each cancer type was not as strongly correlated to the number of oncodomains (Pearson’s Correlation: 0.14) or oncodomain hotspots (Pearson’s Correlation: 0.21), which is to be expected since the number of somatic variants per tumor is known to be highly variable between cancer types [25]. However, the importance of including more sequenced patients for research is highlighted in S1 Table. To address this, a bootstrapping analysis was performed 100 times for the three largest TCGA sets (LUAD, SKCM, and UCEC) to calculate oncodomains and oncodomain hotspots using only 75% and 50% of the available patients and, separately, the available exonic somatic variants. Results for bootstrapping patients or variants both suggest that more oncodomains and oncodomain hotspots will be identified when more data become available, as expected.

We also tested the effect of combining patients from all cancer types to observe whether oncodomains and oncodomain hotspots differ from the cancer-specific hotspots analysis. In this separate analysis, we observe an increase of 82 oncodomains and 1,469 oncodomain hotspots (Pfam only) when combining all data types together that were not identified when analyzing the sets individually (S3 File). Results from the combined dataset also show that 247 oncodomains and 1,251 oncodomain hotspots that were previously identified when analyzing individual datasets are no longer significant in the combined dataset. This, however, is to be expected due to the disproportionate number of patients in each cancer type, removing much of the cancer-specific signals.

### Cancer-specific heterogeneity in oncodomain family somatic mutation rates

**Fig 2:**
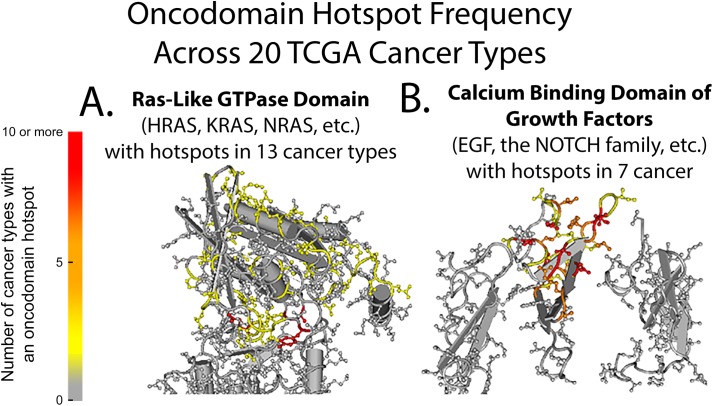
Hotspot frequency of the Ras-like GTPase oncodomain and the calcium binding Epidermal Growth Factor domain. Structural representations of the Ras-like GTPase (cd00882) oncodomain family (A) and the calcium binding domain of the epidermal growth factor-like (cd00054) oncodomain family (B).

Like genes, protein domains have been shown by Nehrt *et al*. and Yang *et al*. to display heterogeneity in the prevalence of somatic variants from patients with different cancer types. However, no study yet has explored the mutation patterns of domain families that appear several times throughout the human genome. In our analysis, we observed this heterogeneity in the prevalence of somatic variants between different cancer types and also between the frequencies in which members of a particular domain family are involved. For example, in S2 File, the hotspots formed on a particular oncodomain are found to be highly heterogeneous in the quantity and location for a given cancer type. Depicted in Fig 2 for the Ras-like GTPase family (Fig 2A) and the calcium binding domain of the Epidermal Growth Factor (Fig 2B), the intensity of color at each residue represents the number of cancer types in which that residue was found to be an oncodomain hotspot across the 20 cancer types. In these structural representations, the frequency or specific location in which somatic variants occur is highly heterogeneous between cancer types, a property that would normally be ignored by traditional region-based analyses that group all positions within a gene or domain region into a single bin when testing for significance.

### Enrichment of functional features, conserved residues, & ontological terms

**Fig 3:**
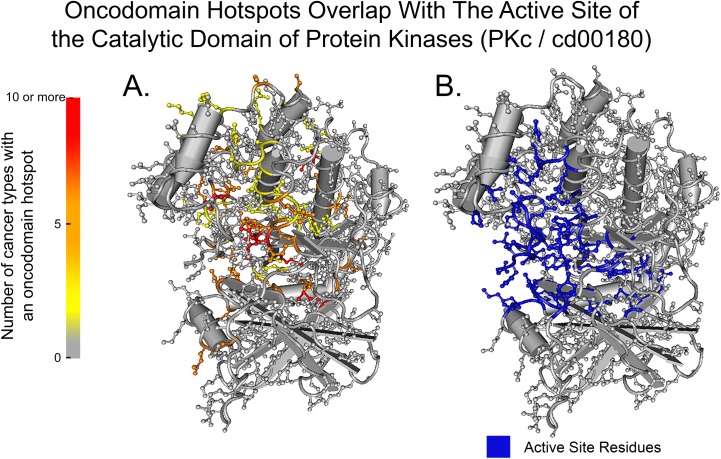
Overlap of oncodomain hotspots with the active site of the catalytic domain of protein kinases. Structural representation of the frequency of oncodomain hotspots across 20 cancer types (A) compared to the active site residues (B) for the PKc / cd00180 oncodomain.

The overlap between oncodomain hotspots and functional features for each protein residue in the UniProt database were ranked by their Fisher’s exact test p-value with Bonferroni correction and are listed in Table 2. Overall, we found that oncodomain hotspots significantly occur on functional feature sites (p-value: 3.63E-87), a finding that is not true for somatic variants overall, which do not occur significantly at functional feature sites (p-value > 0.05). Interestingly, the specific residue of the functional feature that is mutated is heterogeneous between cancer types, as seen in the comparison between the frequency of mutated sites in Fig 3A and the residues involved with the active site in Fig 3B. Additionally, we found a significant overlap between oncodomain hotspots and conserved residues (p-value: 1.45E-09). However, conservation and functional feature annotation do not correlate with oncodomain hotspots (Pearson’s correlation coefficients of 0.009 and 0.048 respectively), indicating that this information alone is insufficient for determining which functional or conserved residues will be important for cancer initiation or progression. For genes with a somatic variant in an oncodomain hotspot, enrichment was performed for categories of genes in the Molecular Function and Biological Process divisions of the Gene Ontology database (S2 Table). For Pfam oncodomains, Gene Ontology term enrichment was performed using the pfam2go annotations (S3 Table).

**Table 2:** Enrichment of residues with functional feature annotation.

Hotspot Enrichment for Functional Feature Annotation on Protein Positions	
Feature Name	P-Value
Nucleotide binding site	2.8E-155
DNA binding site	3.3E-141
Calcium binding site	2.5E-11
Active site	1.7E-7
Metal binding site	3.5E-3

### Comparison to other methods & databases

**Fig 4:**
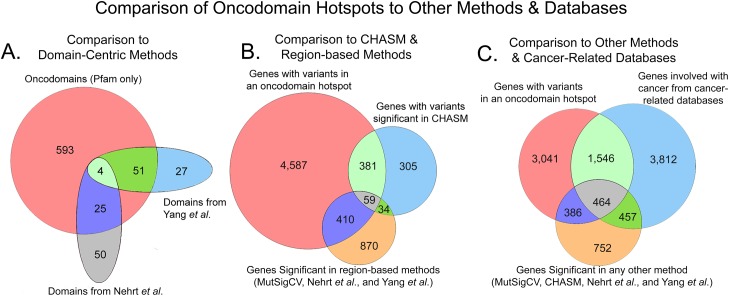
Comparison of oncodomain hotspots to other methods and databases.

**Fig 5:**
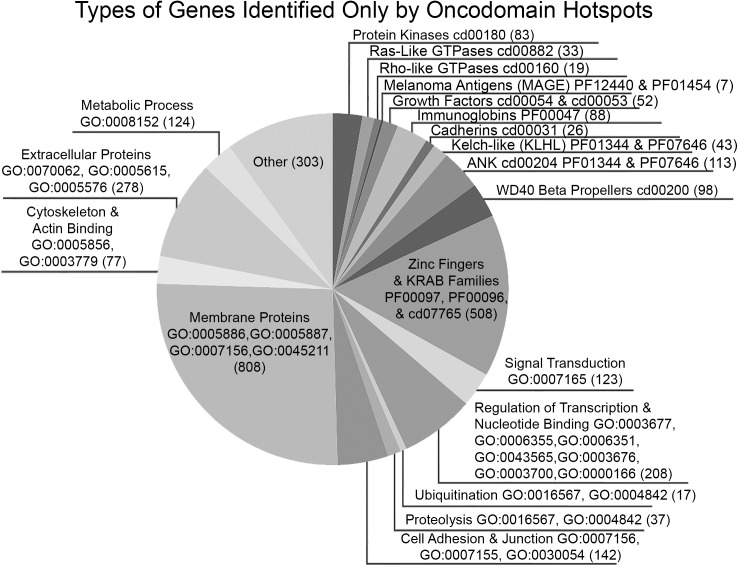
Types of genes identified only by oncodomain hotspots.

Overall, we found that oncodomain hotspots identify more protein domains, genes, and somatic variants than other methods, many of which are rare variants. Due to the lack of a good benchmarking set, we compared the results of our method to the results of other methods for analyzing somatic tumor genomes and to databases of genes with evidence of cancer involvement. In comparison to other domain-centric methods (Nehrt *et al*. and Yang *et al*., Fig 4A), oncodomain hotspots recapitulate 80 / 157 (51%) of Pfam domain models while identifying 593 novel Pfam models. At the gene-level in Fig 4B, genes with variants in an oncodomain hotspot identify 440 / 779 (56%) of genes with variants significant in CHASM, 469 / 1,373 (34%) of genes identified by region-based methods (MutSigCV, Nehrt *et al*., and Yang *et al*.), and 4,587 genes were unique to oncodomain hotspots. Of these 4,587 genes unique to oncodomain hotspots, we found 1,546 / 4,587 (34%) genes to have evidence of cancer involvement from the Cancer Gene Census, the NCI Cancer Gene Index, the Network of Cancer Genes, the Uniprot “proto-oncogene” and “tumor suppressor gene” classifications, and the TSGene databases (Fig 4C) which were not detected by MutSigCV or CHASM. As depicted in Fig 5, the majority of the remaining genes detected only by oncodomain hotspots (2,738 / 3,041; 90%) are either members of domain families for which cancer relevance is known (e.g., kinases, growth factors, and immunoglobins) or are annotated with GO terms that have known cancer relevance (e.g., signal transduction, metabolic process, and cell adhesion).

### Oncodomain hotspots enable the functional analysis of rare somatic variants

Rare variants are thought to play an important role in cancer and, thus, frequency-based methods are inherently ill-suited to assess their relevance in cancer due to their low prevalence in tumor samples. However, by comparing to other genes within the same domain family, oncodomain hotspots have the ability to infer functional relevance of variants that occur infrequently in tumor samples. Indeed, variants implicated only by oncodomain hotspots occurred in an average of 1.1 (variance of 0.34) tumor samples compared to variants implicated by MutSigCV that occurred in an average of 2.1 (variance of 64.4) tumor samples (t-test p-value: 3.5E-259). On the other hand, as expected, oncodomain hotspots implicate many of the frequently occurring variants that would be identified by other methods since the variants in oncodomain hotspots that were also identified by MutSigCV occur in an average of 2.2 (variance of 59.3) tumor samples.

## Discussion

Distinguishing between drivers and passengers in sequenced tumor samples is a challenging task in cancer biology. However, traditional methods that rely solely on frequency of somatic variants for identifying driver variants are limited due to the lack of sequenced patients, even with the thousands of patients that have been sequenced in TCGA. As noted in Sjöblom *et al*. and Wood *et al*., the genomic landscapes of somatic mutations are dominated by “gene hills”, or infrequently mutated genes that do not reach statistical significance but may still be relevant in cancer. Thus, new methods are needed in order to functionally characterize these rare variants and their importance in cancer. As shown in previous studies, Nehrt *et al*. and Yang *et al*., domain-centric analyses have the potential to identify somatic mutational patterns unique to specific cancer types that would normally be overlooked by gene-centric analyses that consider only whole proteins and not the modular regions within. Such approaches can help improve our understanding of the molecular perturbations leading to cancer initiation and progression and enable the identification of new targets for cancer-specific drug research. However, these approaches consider only variation between domain regions within a single gene and, as such, ignore similar, often rare variants in other members of the same protein family that may play a similar role in cancer or may also affect drug treatments. In this study, by leveraging the knowledge of conserved regions of proteins that can occur several times throughout the genome (i.e., protein domains), we are able to infer functional and structural relevance of rare somatic variants by comparing them to similar variants in other genes sharing a common protein domain. This novel concept also allows us to observe heterogeneity in mutation prevalence between members of a protein family—patterns which can be unique for particular cancer types.

In this work, we identify “oncodomain hotspots”, or positions within protein domain regions that harbor more somatic variants than expected by chance by aligning similar domain regions from multiple genes across all patients for a given cancer type (Fig 1). Overall, we found the location and intensity of oncodomain hotspots to be highly heterogeneous between cancer types. For example, as enumerated in S2 File, we found that position five on the Ras-like GTPase (Fig 2A) was the most frequently occurring hotspot on cd00882, appearing in 10 cancer types (BLCA, BRCA, COAD, LUAD, OV, PAAD, READ, SKCM, STAD, and UCEC) and represents a portion of the GTP/M2+ binding site. However, this hotspot was not found in THCA, where oncodomains identified, instead, a hotspot on position 307. Similarly, in LIHC, oncodomain did not identify position five or 307 as hotspots but we reported a hotspot at seven other positions, two of which can only be found in LIHC. Thus, some hotspot patterns are common in several cancers while others are unique to a specific cancer type. In the Ras-like GTPase alone, we find one hotspot unique to COAD, two hotspots unique to LIHC, five hotspots unique to LUAD, six hotspots unique to SKCM, three hotspots unique to STAD, and 20 hotspots unique to UCEC. Interestingly, while we observe a stark heterogeneity between the location and intensity of oncodomain hotspots between cancer types, our results show a significant overlap for oncodomain hotspot location with conserved residues and functional feature sites. Thus, although oncodomain hotspots are heterogeneous, they tend to occur at different positions that are highly conserved residues or at different positions that perform similar functions as seen in Fig 3 where hotspots tend to occur spatially around the active site of the catalytic domain of protein kinases.

Overall, oncodomain hotspots identify many more domains (Fig 4A) than other domain-centric methods like Nehrt *et al*. and Yang *et al*. and more genes (Fig 4B) than gene-centric methods like MutSigCV or CHASM. Although not identified by other methods, 1,546 / 4,629 (34%) of genes identified only by oncodomain hotspots have evidence of cancer involvement from the Cancer Gene Census, the NCI Cancer Gene Index, the Network of Cancer Genes, the Uniprot “proto-oncogene” and “tumor suppressor gene” classifications, and the TSGene manually curated databases (Fig 4C). Interestingly, we find variants in oncodomain hotspots on 392 genes from either the TSGene database or UniProt’s tumor suppressor gene annotations, indicating that both oncogenes and tumor suppressors form hotspots at the domain-level, a phenomenon previously discovered for tumor suppressor genes at the gene-level [67–69]. Moreover, as illustrated in Fig 5, the majority (90%) of the remaining 3,041 genes in Fig 4C identified only by oncodomain hotspots are either members of domain families for which cancer relevance is known or are annotated with GO terms that are known to be important for cancer. Overall, oncodomain hotspots find many new genes that display similar somatic variant patterns to other genes within the same domain family that are well-studied in cancer genomics including 83 novel kinases (cd00180), 52 novel growth factors (cd00054 & cd00053), 33 novel Ras family members (cd00882), 26 novel cadherins (cd00031), 88 novel immunoglobins (pfam00047), and 43 novel Kelch-like (KLHL) genes. Additionally, oncodomain hotspots identify significant somatic variant clusters in the Melanoma Antigen (MAGE) family of genes which were never significant in other methods as well as the Rho-like GTPase family, which has known cancer involvement but is notorious for being somatically mutated only rarely [70,71]. Oncodomain hotspots also identify many genes involved with cell adhesion and cell junction organization, which are known to be important in cancer progression [72–74] and metastasis [75,76], and genes involved with metabolism, which are also important in cancer progression [77–79]. Furthermore, many genes involved with the extracellular matrix or extracellular vesicles formed oncodomain hotpots, which are thought to be important in the regulation of cancer progression and metastasis [80–85]. Oncodomain hotspots are also formed on other gene families involved with processes thought to influence cancer initiation or progression such as ubiquitination [86–88], proteolysis [89–91], metabolic proteins [92,93], and genes involved with actin binding and the cytoskeleton [94–96]. Interestingly, oncodomain hotspots also identify many membrane proteins, which are involved with signal transduction, which is known to be relevant in cancer [97,98] and experimental evidence confirms the important regulatory role played by membrane proteins in cancer [99–105]. Our results also indicate a strong pattern of variants occurring at specific domain family sites for genes involved with signal transduction, regulation of transcription, and nucleotide binding GO terms. Likewise, we find oncodomain hotspots in domain families that serve as the molecular machinery of transcription factors (zinc fingers, KRAB domains, and WD40 beta propellers) as well as ANK domains, which mediate protein-protein interactions [106]. Thus, oncodomain hotspots reveal a vast landscape of somatic variants that act at the level of domain families altering signaling pathways and gene regulation to influence cancer.

**Fig 6:**
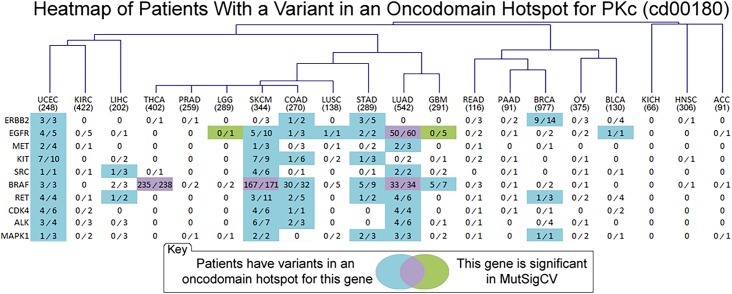
Heatmap of Patients with a Variant in an Oncodomain Hotspot for the PKc domain. Visual representation and hierarchical clustering of oncodomain hotspots on genes that were significant in MutSigCV. For each gene in each cancer type, the number of patients in oncodomain hotspots is quantified and the cell is color-coded if the gene had any patients in oncodomain hotspots (blue), if it was significant in MutSigCV (green) or both (purple). Only the top ten genes based on the gene name’s co-occurrence with the “cancer” MeSH term are shown. Here, cancer types are grouped via hierarchical clustering to show similar mutational patterns. Enumerated in each cell are the proportion of patients with a somatic variant in an oncodomain hotspot (numerator) compared to the number of patients that had a somatic variant anywhere in the protein domain region (denominator).

Identifying the role in cancer, if any, of so-called “gene-hills” in Sjöblom *et al*. and Wood *et al*. has been an important challenge since rare variants are thought to play an important role in cancer [6,107,108], which has led to an increase in network-based analyses for functional characterization [24,27–30]. A domain family-based analysis like oncodomain hotspots enables the identification of many novel, often rare variants that occur more frequently in specific positions within domain families than expected by chance. Indeed, when analyzing entire families of proteins and not specific members therein, mutational patterns emerge which suggest that rare variants play an important role since they often occur on genes with known cancer relevance. For example, protein kinases harbor somatic variants in 3,634/5,848 (62.1%) of the tumors analyzed in this study yet only 27 / 465 human genes mapping to the PKc (cd00180) domain model were considered significant by MutSigCV, 16 of which were significant in only the PAAD cancer type. In Fig 6 and S2 Fig, we summarize the results of comparing MutSigCV and CHASM respectively against oncodomain hotspots to evaluate the ability of these methods to identify rare and common variants relevant to cancer. The genes selected are members of the PKc (cd00180) oncodomain family, the catalytic domain of protein kinases that are the most frequently mentioned in PubMed articles annotated with the “cancer” MeSH term, effectively ranking them by how frequently they are mentioned in the cancer literature. This family contains 465 genes encompassing all serine-threonine, tyrosine, and dual specificity kinases in the human genome. Results in Fig 6 highlight the importance of rare variants in cancer since many genes with known cancer relevance are not reported by MutSigCV (shown in blue). Several instances exist where these MutSigCV and oncodomain hotspots agree (purple) and also where MutSigCV finds significance where the oncodomain method did not (green). Surprisingly, MutSigCV performed poorly for these genes since only two of these genes (*EFGR* and *BRAF*) were significant in MutSigCV for any cancer type. When compared to both MutSigCV and CHASM (S2 Fig), oncodomain hotspots still identify many more variants than MutSigCV and CHASM combined. However, CHASM is a machine learning method and does not incorporate the frequency of the variant but instead utilizes 70 features calculated from properties of genomic and protein sequence, predicted protein structure, and multiple sequence alignments. CHASM’s Random Forest algorithm is trained on a set of known driver mutations as a positive set and synthetically generated passenger mutations as a negative set. Thus, while MutSigCV would not be able to implicate these rare variants due to insufficient population frequency, CHASM uses properties learned from known driver mutations, which often agree with oncodomain hotspots that utilize population frequency alone. Furthermore, we find that oncodomain hotspots are capable of identifying more rare variants in these kinases than other methods while still identifying the obvious variants that occur with high frequency such as *EGFR* in LUAD and *BRAF* in THCA, SKCM, and LUAD. Moreover, oncodomain hotspots are able to identify genes that are known to be associated with particular cancer types where traditional methods may fail. For example the seven genes identified by oncodomain hotspots for COAD (*ERBB2* [109,110], *EGFR* [111,112], *KIT* [113,114], *BRAF* [115–117], *RET* [118,119], *CDK4* [120–122], *ALK* [123–125], and *MAPK1* [126–128]) are reported to have been involved with COAD. Interestingly, all of these genes were found to be mutated in only six or fewer patients with the exception of *BRAF*, which was mutated in 32 patients but was still not identified by MutSigCV or CHASM in S2 Fig. In other examples, the *SRC* gene is a well-known oncogene involved in the PI-3K cascade but no other method is able to detect any significance while oncodomain hotspots identify 8 somatic variants in oncodomain hotspots for LIHC, LUAD, SKCM, and UCEC where some evidence of *SRC*’s role is known [129–131]. Even for genes that were significant in MutSigCV, oncodomain hotspots are more sensitive as they identify those same genes as significant in more cancer types for which they are known to play a role like *BRAF* in STAD [132–134], GBM [135–137], and UCEC [138,139] and *EGFR* in COAD [111,112], STAD [140,141], and SKCM [142–144]. Indicating the ability of oncodomain hotspots to implicate rare variants, 48 variants on these PKc genes that were found in three or fewer tumor samples fell into oncodomain hotspots and five of these variants were found in only a single tumor sample.

To conclude, in sequenced tumor samples, even somatic variants that are known to drive tumor progression can occur with relatively low frequency. Our novel oncodomain method for identifying likely driver variants reveals the structural and functional mutational patterns on conserved protein domains that are unique to each cancer type. This allows us to infer functional importance of even rare somatic variants via inference to somatic variants in other genes sharing a common protein domain. Determining which variants are most important for tumorigenesis will help elucidate the mechanisms driving tumor progression and could ultimately provide a new set of drug targets for families of genes that display similar variation at the structural and functional level. We expect oncodomain hotspots to be an integral tool for assessing novel rare variants in tumor samples, complimenting other existing tools.

## Supporting information

S1 Fig Frequency of oncodomain families across 20 cancer types. Frequency distribution of the number of times pfam oncodomain families form a hotspot in 20 different cancer types.
(TIF)

S2 Fig Heatmap of Patients with a Variant in an Oncodomain Hotspot for the PKc domain. Visual representation and hierarchical clustering of oncodomain hotspots on genes that were significant in CHASM or MutSigCV. For each cell, the ratio of patients with somatic variants in a hotspot to patients with a somatic variant in the domain region is quantified. Each cell is color-coded if the gene had any somatic variants of that cancer type in an oncodomain hotspot (blue), if it was significant in CHASM/MutSigCV (green), or both (purple). Only the top ten genes based on the gene name’s co-occurrence with the “cancer” MeSH term are shown. Here, cancer types are grouped via hierarchical clustering to show similar mutational patterns. Enumerated in each cell are the proportion of patients with a somatic variant in an oncodomain hotspot (numerator) compared to the number of patients that had a somatic variant anywhere in the protein domain region (denominator).
(TIF)

S1 Table Oncodomains and Oncodomain Hotspot Bootstrap Analysis. Bootstrap analysis was performed to count the number of Pfam oncodomains and oncodomain hotspots with only 75% or 50% of the available patients or available exonic somatic variants. The bootstrapping process was repeated 100 times for each cancer type, bootstrap percentage, and local false discovery rate cutoffs.
(DOCX)

S2 Table Gene Ontology Enrichment. Enrichment of the Biological Process and Molecular Function Gene Ontology ontologies for genes with at least one somatic variant in an oncodomain hotspot for any cancer type.
(DOCX)

S3 Table Enrichment of Pfam Gene Ontology (GO) terms with oncodomains. Top twenty enriched Gene Ontology terms with Pfam oncodomains from the pfam2go annotations using Fisher’s exact test with Bonferroni correction.
(DOCX)

S1 File Frequency of oncodomain occurrence across 20 cancer types. (XLSX)

S2 File List of oncodomains and corresponding oncodomain hotspots. (ZIP)

S3 File List of new oncodomains and oncodomain hotspots identified when combining patients from all categories. (XLSX)
