# Supplementary figures and images for: Oncodomains: A protein domain-centric framework for analyzing rare variants in tumor samples
Source: PLoS Comput Biol. 2017 Apr 20;13(4):e1005428. doi: 10.1371/journal.pcbi.1005428 (PMC5398485; doi:10.1371/journal.pcbi.1005428)

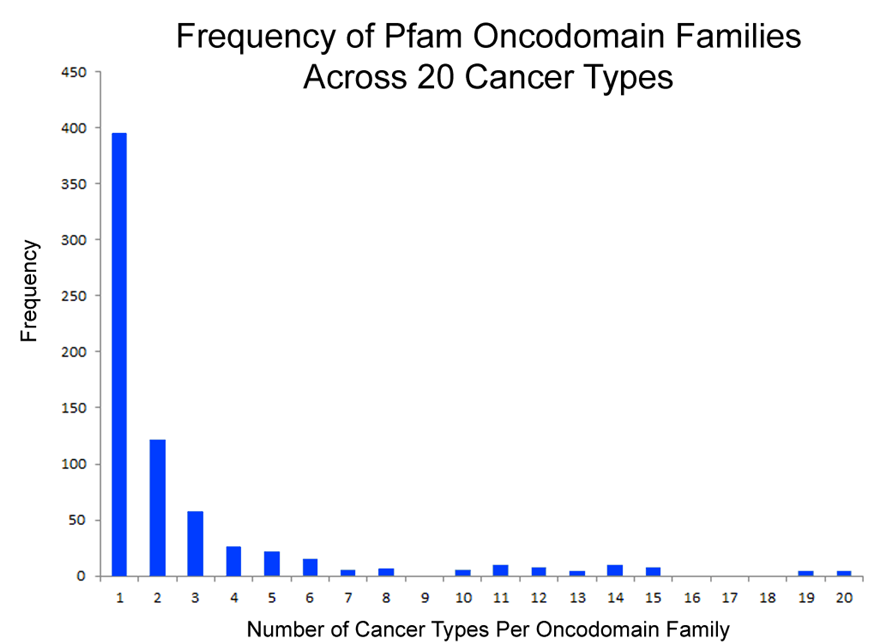

Supplement: S1 Fig — Frequency distribution of the number of times pfam oncodomain families form a hotspot in 20 different cancer types. (TIF) [file pcbi.1005428.s001.tif]

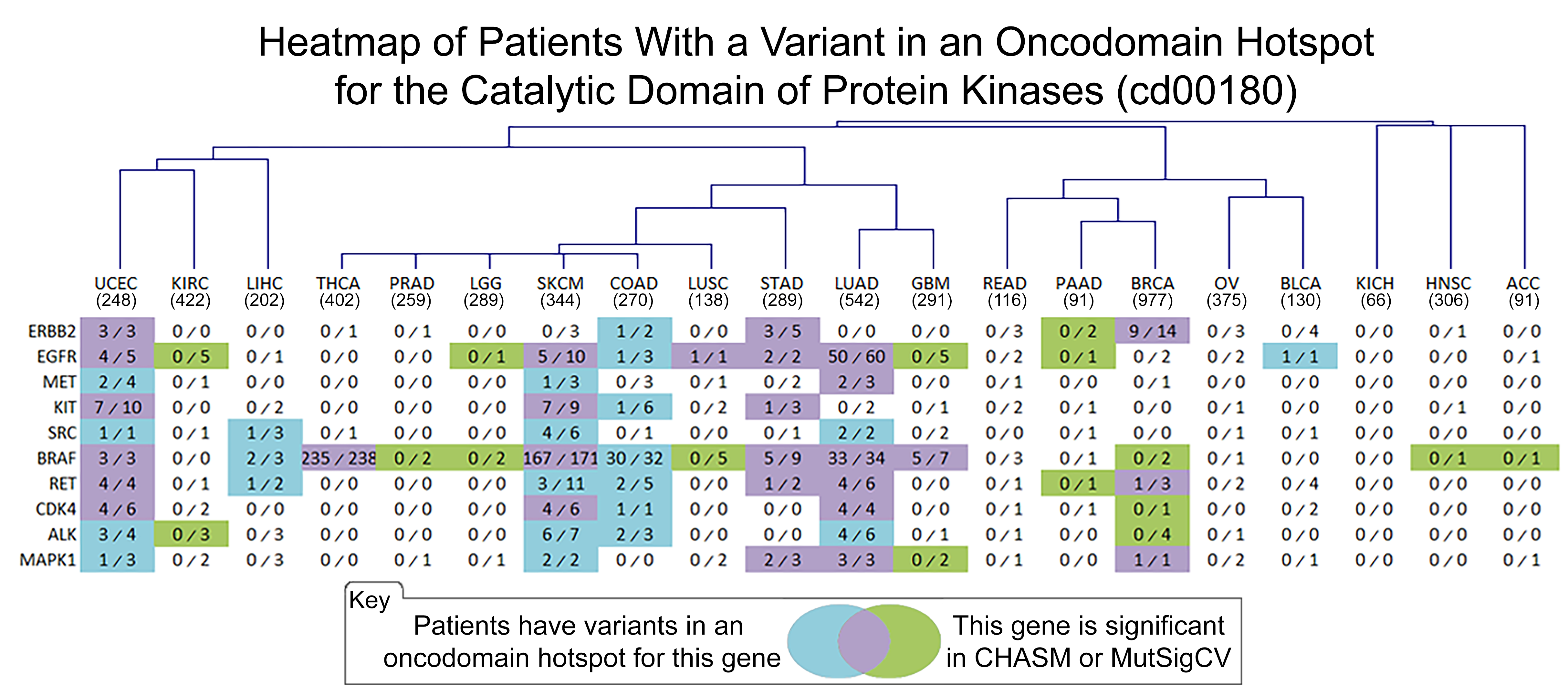

Supplement: S2 Fig — Visual representation and hierarchical clustering of oncodomain hotspots on genes that were significant in CHASM or MutSigCV. For each cell, the ratio of patients with somatic variants in a hotspot to patients with a somatic variant in the domain region is quantified. Each cell is color-coded if the gene had any somatic variants of that cancer type in an oncodomain hotspot (blue), if it was significant in CHASM/MutSigCV (green), or both (purple). Only the top ten genes based on the gene name’s co-occurrence with the “cancer” MeSH term are shown. Here, cancer types are grouped via hierarchical clustering to show similar mutational patterns. Enumerated in each cell are the proportion of patients with a somatic variant in an oncodomain hotspot (numerator) compared to the number of patients that had a somatic variant anywhere in the protein domain region (denominator). (TIF) [file pcbi.1005428.s002.tif]
