# Supplementary material for: Oncodomains: A protein domain-centric framework for analyzing rare variants in tumor samples
Source: PLoS Comput Biol. 2017 Apr 20;13(4):e1005428. doi: 10.1371/journal.pcbi.1005428 (PMC5398485; doi:10.1371/journal.pcbi.1005428)
Supplement: S1 Table — Bootstrap analysis was performed to count the number of Pfam oncodomains and oncodomain hotspots with only 75% or 50% of the available patients or available exonic somatic variants. The bootstrapping process was repeated 100 times for each cancer type, bootstrap percentage, and local false discovery rate cutoffs. (DOCX) [file pcbi.1005428.s003.docx]

**S1 Table: Oncodomains and Oncodomain Hotspot Bootstrap Analysis.** Bootstrap analysis was performed to count the number of Pfam oncodomains and oncodomain hotspots with only 75% or 50% of the available patients or available exonic somatic variants. The bootstrapping process was repeated 100 times for each cancer type, bootstrap percentage, and local false discovery rate cutoffs.

|  | Number of  Pfam  Oncodomains  Using 75% | Number of  Pfam  Oncodomains  Using 50% | Number of  Pfam  Oncodomain Hotspots  Using 75% | Number of  Pfam  Oncodomain Hotspots Using 50% |
| --- | --- | --- | --- | --- |
| Bootstrapping LUAD variants | *fdr*(*t*) = 0.05:  106.8 ± 4.3  *fdr*(*t*) = 0.01:  74.9 ± 4.0 | *fdr*(*t*) = 0.05:  76.6 ± 4.4  *fdr*(*t*) = 0.01:  51.7 ± 3.7 | *fdr*(*t*) = 0.05:  1,331.4 ± 33.5  *fdr*(*t*) = 0.01:  978.0 ± 34.3 | *fdr*(*t*) = 0.05:  957.1 ± 49.8  *fdr*(*t*) = 0.01:  661.0 ± 44.8 |
| Bootstrapping LUAD patients | *fdr*(*t*) = 0.05:  86.8 ± 5.8  *fdr*(*t*) = 0.01:  59.3 ± 4.7 | *fdr*(*t*) = 0.05:  74.6 ± 6.3  *fdr*(*t*) = 0.01:  49.6 ± 4.9 | *fdr*(*t*) = 0.05:  1,040.0 ± 61.9  *fdr*(*t*) = 0.01:  765.0 ± 48.3 | *fdr*(*t*) = 0.05:  882.0 ± 89.7  *fdr*(*t*) = 0.01:  596.2 ± 65.2 |
| Bootstrapping SKCM variants | *fdr*(*t*) = 0.05:  243.2 ± 6.2  *fdr*(*t*) = 0.01:  162.4 ± 6.2 | *fdr*(*t*) = 0.05:  157.9 ± 6.1  *fdr*(*t*) = 0.01:  101.8 ± 5.8 | *fdr*(*t*) = 0.05:  1,498.7 ± 37.3  *fdr*(*t*) = 0.01:  1,032.5 ± 33.5 | *fdr*(*t*) = 0.05:  1,020.4 ± 31.0  *fdr*(*t*) = 0.01:  707.6 ± 36.3 |
| Bootstrapping SKCM patients | *fdr*(*t*) = 0.05:  237.4 ± 19.3  *fdr*(*t*) = 0.01:  158.7 ± 13.5 | *fdr*(*t*) = 0.05:  147.2 ± 20.2  *fdr*(*t*) = 0.01:  94.5 ± 13.6 | *fdr*(*t*) = 0.05:  1,455.8 ± 110.5  *fdr*(*t*) = 0.01:  1,003.4 ± 71.9 | *fdr*(*t*) = 0.05:  949.1 ± 137.9  *fdr*(*t*) = 0.01:  659.7 ± 100.3 |
| Bootstrapping UCEC variants | *fdr*(*t*) = 0.05:  153.1 ± 6.3  *fdr*(*t*) = 0.01:  107.9 ± 5.5 | *fdr*(*t*) = 0.05:  131.6 ± 3.9  *fdr*(*t*) = 0.01:  77.4 ± 4.1 | *fdr*(*t*) = 0.05:  1,217.7 ± 40.9  *fdr*(*t*) = 0.01:  804.3 ± 36.6 | *fdr*(*t*) = 0.05:  941.7 ± 50.0  *fdr*(*t*) = 0.01:  587.1 ± 48.3 |
| Bootstrapping UCEC patients | *fdr*(*t*) = 0.05:  150.5 ± 19.0  *fdr*(*t*) = 0.01:  105.0 ± 11.8 | *fdr*(*t*) = 0.05:  93.9 ± 20.8  *fdr*(*t*) = 0.01:  65.0 ± 15.0 | *fdr*(*t*) = 0.05:  1,183.3 ± 138.8  *fdr*(*t*) = 0.01:  786.7 ± 111.7 | *fdr*(*t*) = 0.05:  660.4 ± 195.5  *fdr*(*t*) = 0.01:  360.8 ± 139.6 |
