# Supplementary material for: Oncodomains: A protein domain-centric framework for analyzing rare variants in tumor samples
Source: PLoS Comput Biol. 2017 Apr 20;13(4):e1005428. doi: 10.1371/journal.pcbi.1005428 (PMC5398485; doi:10.1371/journal.pcbi.1005428)
Supplement: S2 Table — Enrichment of the Biological Process and Molecular Function Gene Ontology ontologies for genes with at least one somatic variant in an oncodomain hotspot for any cancer type. (DOCX) [file pcbi.1005428.s004.docx]

**S2 Table: Gene Ontology Enrichment.** Enrichment of the Biological Process and Molecular Function Gene Ontology ontologies for genes with at least one somatic variant in an oncodomain hotspot for any cancer type.

| **P-Value** | **GO ID** | **GO Term Name** |
| --- | --- | --- |
| 0 | GO:0005515 | protein binding |
| 9.79E-163 | GO:0005886 | plasma membrane |
| 6.31E-145 | GO:0007186 | G-protein coupled receptor signaling pathway |
| 3.03E-142 | GO:0004930 | G-protein coupled receptor activity |
| 1.95E-108 | GO:0004674 | protein serine/threonine kinase activity |
| 4.11E-106 | GO:0006468 | protein phosphorylation |
| 1.58E-101 | GO:0050911 | detection of chemical stimulus involved in sensory perception of smell |
| 1.81E-101 | GO:0004984 | olfactory receptor activity |
| 1.87E-74 | GO:0005887 | integral component of plasma membrane |
| 1.96E-69 | GO:0004672 | protein kinase activity |
| 8.83E-65 | GO:0006351 | transcription, DNA-templated |
| 1.19E-56 | GO:0005524 | ATP binding |
| 4.51E-56 | GO:0003676 | nucleic acid binding |
| 3.13E-55 | GO:0006355 | regulation of transcription, DNA-templated |
| 4.57E-55 | GO:0005509 | calcium ion binding |
