# Supplementary material for: Oncodomains: A protein domain-centric framework for analyzing rare variants in tumor samples
Source: PLoS Comput Biol. 2017 Apr 20;13(4):e1005428. doi: 10.1371/journal.pcbi.1005428 (PMC5398485; doi:10.1371/journal.pcbi.1005428)
Supplement: S3 Table — Top twenty enriched Gene Ontology terms with Pfam oncodomains from the pfam2go annotations using Fisher’s exact test with Bonferroni correction. (DOCX) [file pcbi.1005428.s005.docx]

**S3 Table: Enrichment of Pfam Gene Ontology (GO) terms with oncodomains.** Top twenty enriched Gene Ontology terms with Pfam oncodomains from the pfam2go annotations using Fisher’s exact test with Bonferroni correction.

| **GO Term Description** | **GO Accession** | **P-Value** |
| --- | --- | --- |
| GO:membrane | GO:0016020 | 5.41E-15 |
| GO:protein binding | GO:0005515 | 3.16E-13 |
| GO:signal transduction | GO:0007165 | 2.43E-11 |
| GO:zinc ion binding | GO:0008270 | 7.40E-11 |
| GO:G-protein coupled receptor signaling pathway | GO:0007186 | 9.26E-11 |
| GO:regulation of transcription, DNA-templated | GO:0006355 | 2.42E-10 |
| GO:calcium ion binding | GO:0005509 | 1.06E-09 |
| GO:transmembrane transport | GO:0055085 | 2.17E-09 |
| GO:G-protein coupled receptor activity | GO:0004930 | 4.18E-09 |
| GO:integral component of membrane | GO:0016021 | 1.10E-08 |
| GO:nucleus | GO:0005634 | 1.36E-08 |
| GO:sequence-specific DNA binding transcription factor activity | GO:0003700 | 2.21E-08 |
| GO:GTP binding | GO:0005525 | 9.84E-08 |
| GO:oxidoreductase activity | GO:0016491 | 2.20E-06 |
| GO:proteolysis | GO:0006508 | 3.22E-06 |
| GO:ATP binding | GO:0005524 | 5.12E-06 |
| GO:metabolic process | GO:0008152 | 1.89E-05 |
| GO:metalloendopeptidase activity | GO:0004222 | 2.30E-05 |
| GO:intracellular signal transduction | GO:0035556 | 3.79E-05 |
| GO:transport | GO:0006810 | 0.000194 |
